# Editorial: Engineering Applications of Neurocomputing

**DOI:** 10.3389/fnbot.2022.839505

**Published:** 2022-01-28

**Authors:** Long Wang, Zhe Song, Zijun Zhang, Chao Huang

**Affiliations:** ^1^School of Computer and Communication Engineering, University of Science and Technology Beijing, Beijing, China; ^2^School of Business, Nanjing University, Nanjing, China; ^3^School of Data Science, City University of Hong Kong, Kowloon, Hong Kong SAR, China; ^4^State Key Laboratory of Internet of Things for Smart City, University of Macau, Macau, China

**Keywords:** neurocomputing, engineering systems, neural networks, manufacturing, healthcare

Inspired by how the human brain works, neurocomputing algorithms, including deep learning, reinforcement learning, and neurodynamic optimization, have achieved tremendous success in various applications across many domains, e.g., visual object tracking, speech recognition, human-level control, text understanding, and real-time optimization.

Various types of intelligent equipment and hardware devices have been developed to implement neurocomputing models for engineering systems. Deep learning has been employed for industrial robotic applications, including stereo reconstruction, object pose recognition, and product quality check. With the advent of the Internet of Things and edge computing devices, deep reinforcement learning has become popular in the predictive maintenance of engineering equipment. Embedded convolutional neural networks are widely utilized for autonomous vehicle control. The success of applying neurocomputing approaches and related hardware implementations in different engineering domains, such as intelligent manufacturing, energy internet, and smart healthcare, has proven the potential of employing neurocomputing for solving real problems in various engineering fields.

In recent years advances in sensor and data storage technologies have enabled the accumulation of a large amount of data from engineering systems. Driven by big data generated from engineering systems, neurocomputing, and its hardware implementation will continually transform engineering systems into more intelligent forms.

This Research Topic aims to provide a forum for researchers to present the latest research on applications of neurocomputing algorithms and neurocomputing-based hardware in engineering systems. It brings together 14 high quality papers reporting on the latest applications of neurocomputing in transportation, manufacturing, biomedical engineering, electrical engineering, and knowledge management.

In the paper entitled “*Self-Triggered Consensus of Vehicle Platoon System With Time-Varying Topology*,” Wang et al. designed a secure event-triggered controller considering the safe distance for the vehicle platoon system. Based on the new event-triggered function, a more energy efficient self-triggered control strategy was developed by using the Taylor formula. The new self-triggered control strategy could avoid continuous calculation and measurement of vehicles.

The paper on “*Industrial Control Malicious Traffic Anomaly Detection System Based on Deep Autoencoder*” by Wang et al. proposes a method of detecting abnormal traffic in industrial control networks based on autoencoder technology. The Kullback–Leibler divergence was added to the loss function of the proposed model to improve the ability of feature extraction and the ability to recover raw data. The gas pipeline dataset was used to verify the performance of the proposed method.

In a contribution on “*Data-Driven Hybrid Equivalent Dynamic Modeling of Multiple Photovoltaic Power Stations Based on Ensemble Gated Recurrent Unit*” Long et al. report on a data-driven hybrid equivalent model for the dynamic response process of the multiple PV power stations. The data-driven hybrid equivalent model contained the simple equivalent model and data-driven error correction model. The simulation results validated the super-performance of the proposed model both in response speed and accuracy.

Sun et al. contribute a paper on “*AutoPath: Image-Specific Inference for 3D Segmentation*,” which introduces *AutoPath*, an image-specific inference approach for more efficient 3D segmentations. The proposed AutoPath dynamically selected enabled residual blocks regarding different input images during inference, thus effectively reducing total computation without degrading segmentation performance. Experimental results on a liver CT dataset showed that the proposed approach not only provided an efficient inference procedure but also attained satisfactory segmentation performance.

A paper on “*Boosting Knowledge Base Automatically via Few-Shot Relation Classification*” by Pang et al. investigated a fully automatic method to train a relation classification model which facilitates to boost the knowledge base. In the proposed method, various multiple instance learning methods were incorporated into the classic prototypical networks, reducing sentence-level noises.

Gu et al.'s article “*Investigating the Impact of the Missing Significant Objects in Scene Recognition Using Multivariate Pattern Analysis*” adopted multivariate pattern analysis to explore the object-scene association in scene recognition when different amounts of significant objects were masked. The analysis results suggested that the lateral occipital complex was sensitive to the loss of significant objects and mainly involved in scene recognition by the object-scene semantic association.

In the paper “*Machine Learning Models to Predict Primary Sites of Metastatic Cervical Carcinoma From Unknown Primary*,” Lu et al. conducted a series of bioinformatics analyses based on a dataset from The Cancer Genome Atlas (TCGA) RNA-Seq data of squamous cancer and TCGA Pan-Cancer data. Three machine learning models, random forest, neural networks, and support vector machine, were developed to explore potentially effective tools to distinguish these squamous cancers.

In their contribution examining “*A Manufacturing-Oriented Intelligent Vision System Based on Deep Neural Network for Object Recognition and 6D Pose Estimation*” Liang et al. present a new two-stage intelligent vision system based on a deep neural network with RGB-D image inputs for object recognition and 6D pose estimation. A dense-connected network fusing multi-scale features was first built to segment the objects from the background. The 2D pixels and 3D points in cropped object regions were then fed into a pose estimation network to make object pose predictions based on the fusion of color and geometry features.

The paper titled “*Artificial Intelligence-Based Application to Explore Inhibitors of Neurodegenerative Diseases*” by Deng et al. explores an integrated new approach for finding lead compounds that inhibit galectin-3, by combining universal artificial intelligence algorithms with traditional drug screening methods. Manifold artificial intelligence algorithms were performed to validate the docking results and further screen compounds.

In their “*Energy Investment Risk Assessment for Nations Via Seq2seq Model*” Liang et al. propose a sequence to sequence model to evaluate the energy investment risk of 50 countries. Bi-long-short term memory was used as an encoder to process the historical statistics in the proposed method.

The paper on “*A Relational Adaptive Neural Model for Joint Entity and Relation Extraction*” by Duan et al. describes a relational-adaptive entity relation joint extraction model based on multi-head self-attention and densely connected graph convolution network. In the model, the multi-head attention mechanism was specifically used to assign weights to multiple relation types among entities to ensure that the probability space of multiple relations was not mutually exclusive.

An article on the “*Classification of Metastatic and Non-Metastatic Thoracic Lymph Nodes in Lung Cancer Patients Based on Dielectric Properties Using Adaptive Probabilistic Neural Networks*” by Lu et al. proposes a classifier to identify metastatic and non-metastatic thoracic lymph nodes in lung cancer patients based on dielectric properties. Compared with the other methods, the proposed classifier achieved a higher classification accuracy based on dielectric property data collected from lung cancer patients.

Xiang et al.'s contribution on the “*Prediction of Dangerous Driving Behavior Based on Vehicle Motion State and Passenger Feeling Using Cloud Model and Elman Neural Network*” presentes a new method for dangerous driving behavior prediction using a hybrid model consisting of cloud model and Elman neural network based on vehicle motion state estimation and passenger's subjective feeling scores.

The paper titled “*A New Way of Airline Traffic Prediction Based on GCN-LSTM*” by Yu employed graph convolutional neural network and long short memory network to construct an airline traffic prediction system with short-term prediction ability.

These successful applications of neurocomputing in various domains demonstrate the significant potential of applying neurocomputing approaches to solving complex engineering problems. With the help of big data and increasing computing power, neurocomputing will play a vital role in future engineering systems.

## Author Contributions

LW wrote the manuscript. ZZ, ZS, and CH edited the manuscript. All authors contributed to the article and approved the submitted version.

## Funding

This work was supported in part by the National Natural Science Foundation of China under Grant 62002016, in part by the Guangdong Basic and Applied Basic Research Foundation under Grants 2020A1515110431 and 2019A1515111165, and in part by the Fundamental Research Funds for the Central Universities and the Youth Teacher International Exchange & Growth Program under Grant QNXM20210037.

## Conflict of Interest

The authors declare that the research was conducted in the absence of any commercial or financial relationships that could be construed as a potential conflict of interest.

## Publisher's Note

All claims expressed in this article are solely those of the authors and do not necessarily represent those of their affiliated organizations, or those of the publisher, the editors and the reviewers. Any product that may be evaluated in this article, or claim that may be made by its manufacturer, is not guaranteed or endorsed by the publisher.

